# Smart Hospital Sensor Network Deployment for Mobile and Remote Healthcare System 

**DOI:** 10.3390/s21165514

**Published:** 2021-08-17

**Authors:** Yoonkyung Jang, Intae Ryoo, Seokhoon Kim

**Affiliations:** 1 Department of Computer Science and Engineering, Kyung Hee University, Yongin 17104, Korea; jokjjs0216@khu.ac.kr; 2 Department of Software Convergence, Soonchunhyang University, Asan 31538, Korea; 3 Department of Computer Software Engineering, Soonchunhyang University, Asan 31538, Korea

**Keywords:** hospital sensor network, network operation cost, group numbers, optimal gateway deployment

## Abstract

In this paper, we propose a hospital sensor network deployment method for smart healthcare systems. Since sensor nodes in hospitals are always in an environment where power can be supplied, it is essential to have stable network connectivity by achieving optimal gateway deployment, rather than focusing on energy efficiency. The proposed technique leads to an access point (AP) layout that minimizes the overall network operation cost. The operation cost is calculated per unit time, and it includes installation cost and maintenance cost. In addition, group numbers are assigned to sensor nodes for guaranteeing network connectivity, no matter where the mobile sensor devices move. The performance of the proposed methodology has been verified through numerical experiments.

## 1. Introduction

Wireless sensor networks (WSNs) change people’s way of life and are convenient in various fields, such as environment, transportation, industry and so on [1]. For example, WSNs are imported as road information providers to detect various means of transportation and analyze the traffic flow [2]. Additionally, smart factory extends the lifetime of utility machines by checking for noise, illegal vibration, and high temperatures, using WSNs without direct human intervention [3]. Furthermore, WSNs in hospitals improve quality of life by negating wires, i.e., going wireless, and will be enhanced the mobility and stability of medical devices. It also increases the efficiency of patients who need to use wireless devices permanently and offers affordable medical solutions [4]. As the elderly population increases and an aging society approaches, interest in the healthcare system is increasing. The Population Reference Bureau predicted that over the next 20 years, the number of people aged 65 and older will increase by 20% worldwide [5,6].

IoT networking technology is used in many fields such as healthcare services, hospital healthcare monitoring systems, and medical research. Continuous monitoring of patient medical information enables the early detection of emergencies and diseases [7]. It provides remote medical treatment and high-quality medical services to vulnerable areas by establishing an IoT medical networking infrastructure [8]. Additionally, it is possible to take necessary measures by communicating the status of emergency patients being transferred to doctors by ambulance [9]. In addition, patients who need continuous management can measure blood pressure, heart rate, etc., without actually visiting a hospital and can transmit the results to a doctor remotely [10].

In addition, in a real hospital, especially since a large number of devices need to be connected to the network, IoT networking technology is essential. In hospitals, all information is electronically managed. The number of sensor devices for hospital healthcare monitoring systems continues to increase [11]. The various sensors were attached [12] to patient bodies for medical information measurement. There are many medical sensors, from small ones, such as measuring the number of steps and heart rate, to devices that measure the condition of severely ill patients [13]. Instead of paper, doctors and nurses carry tablets and laptops to treat patients [1].

In addition, most people, including medical staff, patients, and carers, have their own personal smart devices. Therefore, a hospital must deal with numerous sensors, ranging from medical devices to personal devices. A hospital is a network environment in which a number of motion sensors and fixed sensors are mixed.

In particular, since hospitals are made up of many hospital rooms, there are many obstacles to transmit data. According to Table 1, the signal strength of a gateway in an indoor environment is affected by distance and obstacles. If the devices sending and receiving data are in different rooms, there is a risk of losing data [14].

A large amount of information in hospitals should be delivered through a stable and reliable network and there should be no delay in data delivery. Delays in data transfer can seriously affect a patient’s life [15]. Nevertheless, most medical institutions use the existing communication infrastructure to transmit medical information. However, the existing communication infrastructure is unstable and has many limitations in handling mobile sensors [16,17]. Additionally, the number of sensors used in hospitals is increasing; there may be a shortage of available bandwidth [18,19].

The access point (AP) is deployed without considering the characteristics of the hospital. Unlike general buildings, hospitals have multiple patients in one room. Because there are not only patients but also medical staff and guardians, the density of people is higher than in general buildings. In a general building, one person uses one or two wireless devices that need to be connected to the Internet, such as cell phones and laptops. However, in a hospital, personal wireless devices, medical devices, and sensors for monitoring the patient’s biometric information need to be connected to the Internet. A patient’s biometric information is time-critical and must be processed quickly because it has a lot to do with the patient’s life. Since APs are installed in consideration of only basic mobile devices before electronically managing medical information, it is difficult to handle medical information quickly and cost-effectively. It is required that AP locations and communication methods are suitable for the hospital.

Original sensor networks are designed with the goal of minimizing energy consumption. However, since hospitals can always supply electricity, a sensor network focused on enabling the rapid and accurate medical treatment, rather than consuming less energy, is needed [20,21].

In this paper, we propose a sensor network for stably serving medical information. A ‘deployment reference’ is created to find suitable AP deployment locations for a hospital and, based on this, the entire sensor network is divided. The ‘deployment reference’ includes the location of gateways that minimizes the installation cost and maintenance cost for each number of sensors. In the first division step, the entire network is divided based on communication range. The optimal gateway placement position is found in the second division step by dividing it (based on the installation cost). In the third division step, if one gateway is in charge of a sensor node greater than a certain standard, the additional gateway is placed in the corresponding area as a result of completing the second division step.

After AP deployment, each sensor is assigned a group number. Even if the sensor moves away from the AP, communication is possible using this group number. Each sensor delivers data to its next higher layer, and the data is ultimately delivered to the AP. A new group number is assigned when the sensor moves and communicates with the existing group number is no longer possible.

The rest of this paper is organized as follows. In Section 2, we provide the AP configuration of the origin hospital network, and the reason for the proposal in this paper is shown. The proposed method for AP deployment is described in Section 3. The proposed method for assigning group numbers to sensor nodes is described in Section 4. In Section 5, we compare the suggested method in this paper and the method for comparing all cases through simulations. Finally, in Section 6, we complete the paper with a conclusion and discussion.

## 2. Original Hospital Network

**Figure 1:**
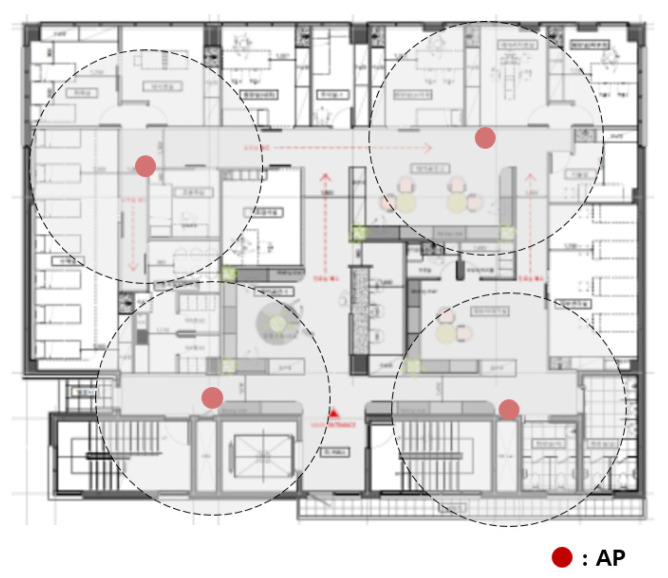
Original hospital APs.

The original hospital APs were installed sporadically, as shown in Figure 1. The APs currently in use were installed before medical information was electronified. Wireless devices are used by being directly connected to the nearest AP, and sending data was not possible if they were located outside the communication range of the AP.

**Figure 2:**
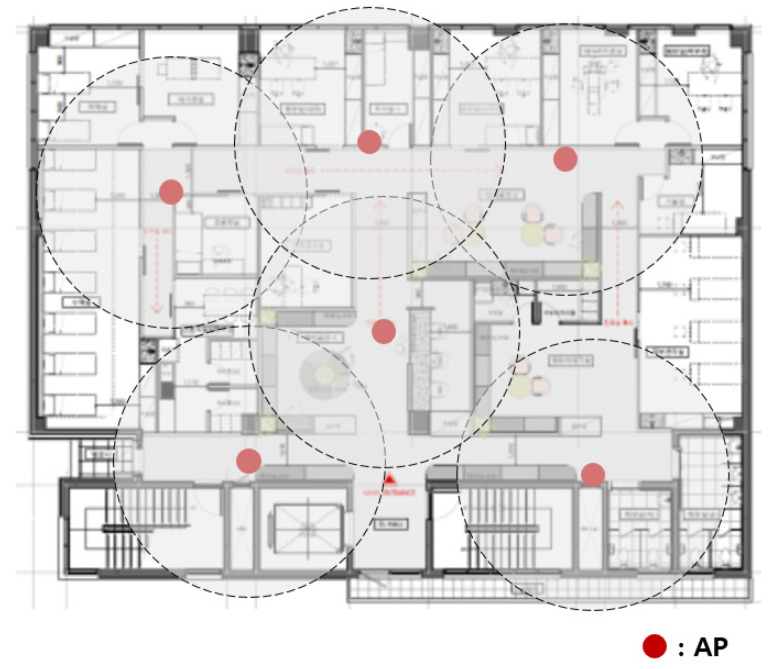
Adding APs to the original hospital network.

The more wireless medical sensors connected to the more APs needed. In this case, a new AP was installed sporadically where necessary. Figure 2 shows the adding of APs to the network of Figure 1. A network connection was possible around the newly installed AP, but another communication blind spot occurred. Intuitively installed APs were inefficient in terms of cost.

LOBIN is a healthcare IT platform that monitors ECG results, heart rate, and tracking location of patients; it uses e-textile fabrics in patient clothing and provides versatile IT infrastructure [22]. However, it does not take into account the mobile usage of doctors, nurses, and caregivers.

The hospital asset tracking method calculates the communication range of a sensor node using a formula for the area of a circle. If there is a wall, it is assumed that the signal from the sensor node does not reach the space behind the wall. Therefore, the whole hospital floor is divided into a grid pattern, and APs are placed so that communication is possible in all spaces [23]. However, although communication is not always impossible in the space behind the wall, this method has the disadvantage of unnecessarily installing APs space behind the wall.

In this paper, we propose a network for stable medical information transfer. It considers the network usage of doctors, nurses, patients, and caregivers, as well as the network usage of hospital devices. Additionally, since the proposed method measures the communicable range, based on whether the signal is transmitted or not, the AP can be installed rationally in the space behind the wall. First, we propose a method for finding the location of the APs and cost-effectively controlling numerous wireless devices in the hospital. APs are more expensive to install and maintain than general sensors. It is necessary to deploy the smallest number of APs to produce the best cost-effective results. 

Second, we propose a group number-based WSN that can relay data to the AP without being directly connected to the AP. Fixed and mobile sensors deployed in medical sensor systems are grouped based on the distance from AP. The sensors transmit data only to their next higher group. As a result, they transmit data to the AP. When a sensor node cannot transmit data to the next higher group sensor node, a new group number is assigned, and data is transmitted using a new path. This allows sensor nodes that cannot communicate directly with an AP to transmit data.

## 3. Deploying AP with Divide and Conquer Algorithm

The original AP deployment is inefficient for the overall network operation. Installing an AP costs more than installing a normal node. This leads to more installation costs than necessary. In this paper, to find the locations that APs can be installed, the entire hospital’s network is divided into two stages. The first dividing step is based on the available communication distance. In the second dividing step, the optimal sink arrangement location is found by dividing based on the installation cost.

### 3.1. First Dividing Step

In the first dividing step, the entire network is divided based on communication range. The AP must be set individually if the sensor node cannot communicate with the AP or another sensor node. The communication range is calculated based on fixed nodes, not nodes with mobility. Moving nodes cannot be used to calculate communication range because its position changes every time.

A hospital has multiple rooms on one floor, including a hospital room, a medical staff workstation, a lounge, a machine room, and so on. Hospital rooms, medical staff workstations, and restrooms are frequently accessed by moving nodes, and the nodes are concentrated. On the other hand, the machine room is not well accessible by mobile nodes and is often far from the hospital’s center.

**Figure 3:**
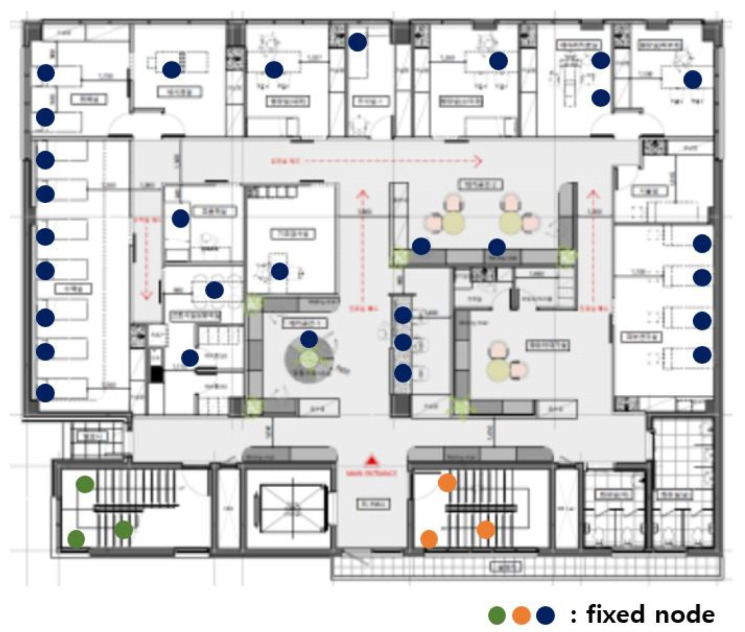
Fixed nodes in the hospital network.

Figure 3 shows the fixed nodes in the hospital network. The green, orange, and blue circles are the fixed nodes with no mobility. Green, orange, and blue nodes have no difference in practical use, but they are expressed in different colors for the readability of the figure. In Figure 3, the green and orange nodes are in machine rooms, so there is no access to mobile nodes, and they are far from the center of the hospital. The green nodes are far from the blue and orange nodes, so communication is impossible. The orange nodes are far from the blue and orange nodes, so communication is also impossible.

**Figure 4:**
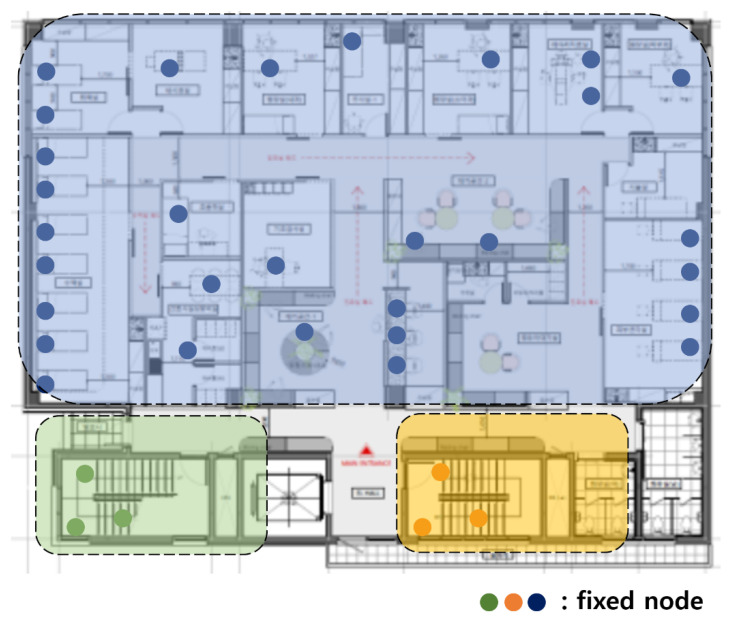
After the first dividing step.

The first dividing step, which divides the network based on communication range, is divided into blue, green, and orange parts. Figure 3 is divided, as shown in Figure 4.

### 3.2. Second Dividing Step

In the second dividing step, the network (that has passed the first dividing step) is divided more, based on cost. Thus, there are 2-1 dividing, 2-2 dividing, and 2-3 dividing steps.

#### 3.2.1. 2-1 Dividing Step

The 2-1 dividing step generates a ‘cost reference’. The ‘cost reference’ is information that includes the number of all topologies that can be generated for each number of sensor nodes. It also has the location of the AP that minimizes the installation cost in each topology.

(1) $$cost\left(x,y\right)=\sum_{i=1}^{n}f\left(\alpha_{x_{i}}\right)\times a+\sum_{i=1}^{m}f\left(\beta_{y_{i}}\right)\times b$$

The cost is calculated by Equation (1); the cost includes the installation and maintenance costs. The *x* is the AP, and the *n* is the number of APs. The *y* is the sensor node and *m* is the number of sensor nodes. The *a* is the installation cost of the AP, and the *b* is the installation cost of the sensor node. Additionally, *f*(*α*) is the number of hops from each sensor node to its assigned AP and *f*(*β*) is the number of hops from each sensor node to the other sensor nodes. Because more energy is consumed, as data is transmitted through more hops, the number of hops is used as a cost calculation factor.

The ‘*p*’ represents the capacity of the AP; ‘*p*’ is set as the maximum number of sensor nodes to be used as a cost reference. The cost reference contains information from one sensor node to ‘*p*’ sensor node.

**Figure 5:**
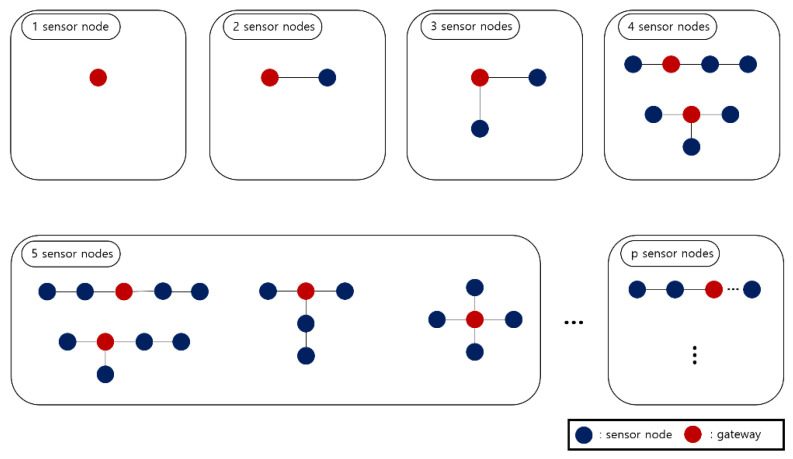
Cost reference.

Figure 5 is the cost reference. The blue circle represents a normal sensor node, and the red circle represents an AP. The cost reference holds all cases for all network topologies that can be created from one to two, three, etc., p sensor nodes. In each case, it also has information on the location of the AP with the minimum cost. Topologies that have the same shape when rotating are not included in the cost reference.

In Figure 5, there is no other option than the sensor node acting as an AP in the case of one sensor node. In the case of two sensor nodes, the total number of topologies is one and there is no difference in cost, even if any one of them is designated as an AP. In the case of three sensor nodes, the total number of topology cases is one, and it is optimal that the most central sensor node is designated as an AP. Finally, in the case of four sensor nodes, the total number of topologies is two, and a red circle indicates optimal AP location for each topology. In this way, the cost reference includes the number of all topology cases from one to *p* sensor nodes, and information on the optimal AP location for each number of cases. 

#### 3.2.2. 2-2 Dividing Step

The 2-2 dividing step finds a combination of sensor nodes, constituting the entire network topology. Since the sensor nodes that have gone through the first dividing step are connected linearly, the sum of the costs, calculated by dividing, is equal to the total cost.

When finding combinations, by dividing the topology, the maximum number of sensor nodes for each combination cannot exceed *p*. This is because the maximum number of sensor nodes that one AP can accommodate is *p*. When the maximum number of APs is ‘*q*’, the total number of combinations constituting the topology cannot exceed *q*. Since sensor nodes with mobility are constantly changing their positions, the second dividing step is performed for fixed nodes. 

In the blue part of Figure 4, there are 30 fixed nodes. When *p* is 9 and *q* is 10, for example, there are many cases in which 30 sensor nodes are divided. It can be divided into combinations, including 9, that maximize the capacity of the AP, such as (9, 9, 9, 1, 1, 1), (9, 9, 9, 1, 2), (9, 9, 9, 3), (9, 9, 8, 1, 1, 2), and (9, 8, 8, 1, 2, 2). It can be divided into combinations containing 8 as the maximum number, such as (8, 8, 8, 1, 1, 1, 1, 1, 1), (8, 8, 8, 2, 1, 1, 1, 1), and (8, 8, 8, 2, 2, 1, 1). In the most simple way, it can be divided into 10 combinations with 3 sensor nodes, such as (3, 3, 3, 3, 3, 3, 3, 3, 3, 3). The number of cases can be varied, within satisfying *p* and *q*. If *p* and *q* are different, the number of combinations is different. The total cost for each case can be obtained by referring to the cost reference that was obtained in the 2-1 dividing step.

Among the obtained combinations, the case that has the lowest cost is assigned to the network topology. This is the process of finding the one that minimizes the cost, among combinations that can satisfy the shape of the entire network. If there is an isolated sensor node (in the result of substitution according to combination), the combination is modified and applied. If there is an isolated sensor node, the sensor node must be included.

(2) a=10, b=3, p=9, q=10.

When the parameters for Equation (1) are set as (2), the result of applying the 2-2 dividing step to the blue part of Figure 4 is in Table 2. The left part of Table 2 is the combinations and cost before applying them to the blue part of Figure 4, and the right part of Table 2 is the combinations and cost after applying them to the blue part of Figure 4. Table 2 is sorted by the cost that calculated before applied.

**Figure 6:**
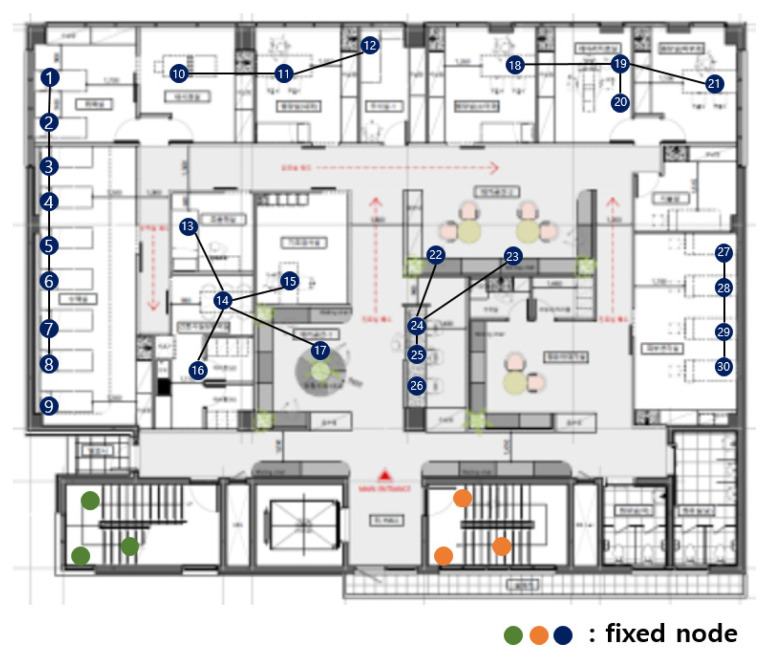
Applying (8, 5, 4, 4, 4, 3, 1, 1).

If the lowest cost (8, 5, 4, 4, 4, 3, 1, 1) is applied, it is as shown in Figure 6. Dividing is conducted sequentially from the largest number. In this case, nodes 9 and 26 are isolated. If the fixed node cannot connect to AP, transfer data is impossible. Therefore, in this case, isolated nodes are included the closest combination. 

**Figure 7:**
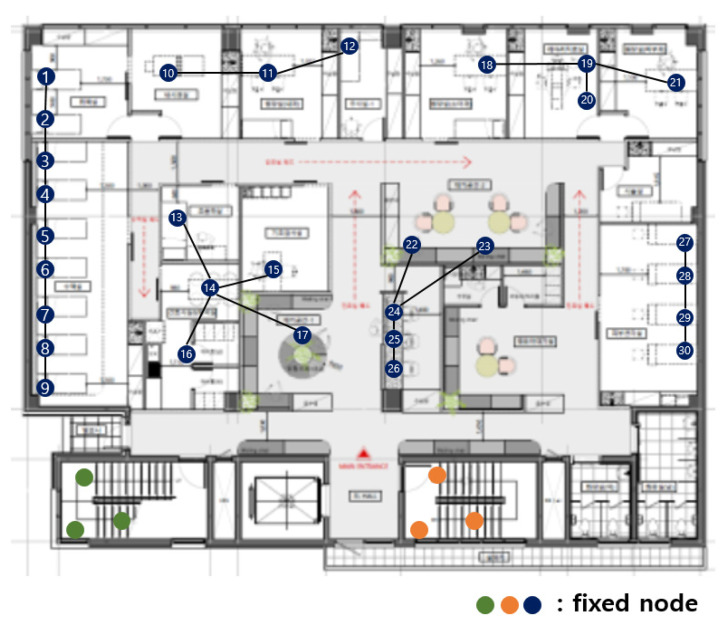
The result of including isolated nodes of the first combination.

Figure 7 shows the result of including isolated nodes 9 and 26. It is divided into (9, 5, 5, 4, 4, 3), and the current cost is higher than the cost, including the isolated node before. The combination of (9, 5, 5, 4, 4, 3) has a higher cost than (8, 8, 4, 4, 3, 3) in the second row of Table 1, so (8, 8, 4, 4, 3, 3) should also be applied.

**Figure 8:**
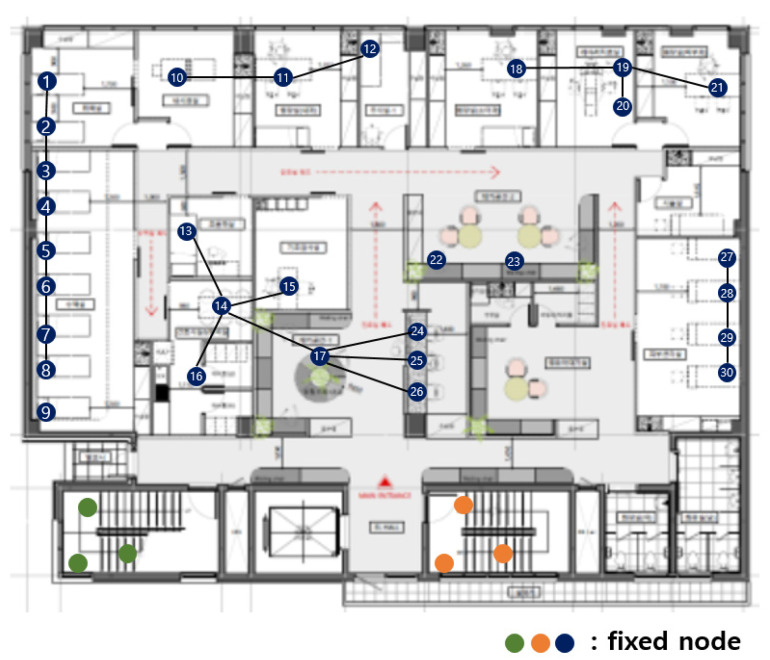
Applying (8, 8, 4, 4, 3, 3).

If (8, 8, 4, 4, 3, 3) is applied, as shown in Figure 8. Dividing is conducted sequentially from the largest number. In this case, nodes 9, 22, and 23 are isolated. Therefore, in this case, isolated nodes are included the closest combination.

**Figure 9:**
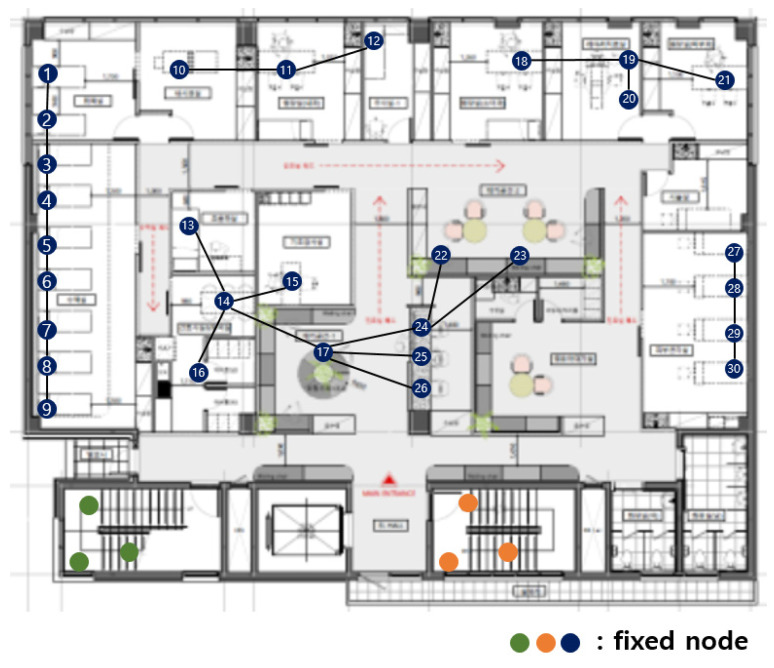
The result of including isolated nodes of the second combination.

Figure 9 shows the result of including isolated nodes 9, 22, and 23. It is divided into (10, 9, 4, 4, 3). Although the cost is higher than the combination before including the isolated node, the cost is lower than (8, 8, 7, 3, 2, 1, 1) in the third row of Table 1, so finally (10, 9, 4, 4, 3) is selected.

When the parameters for Equation (1) are set as (2), the result of applying the 2-2 dividing step to the green part and the orange part of Figure 4 is in Table 3. The left part of Table 3 is the combinations and cost before applying it to the green part and the orange part of Figure 4; and the right part of Table 3 is the combinations and cost after applying it to the green part of Figure 4. Table 3 is sorted by the cost that was calculated before applied.

**Figure 10:**
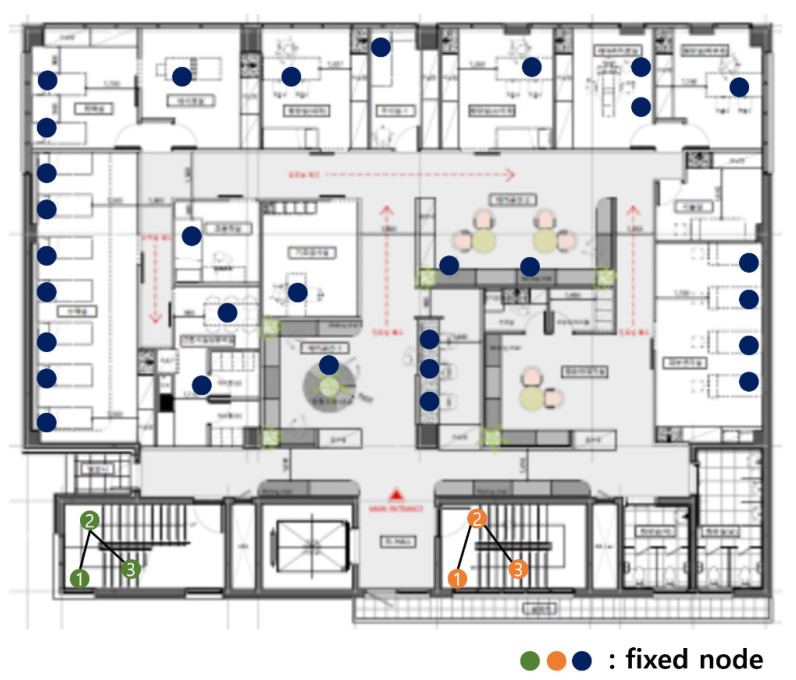
Result of applying the first combination.

If the lowest cost (3) is applied, it is as shown in Figure 10. Since the cost is lower than (2, 1) in the second row of Table 3, the (3) is finally selected.

**Figure 11:**
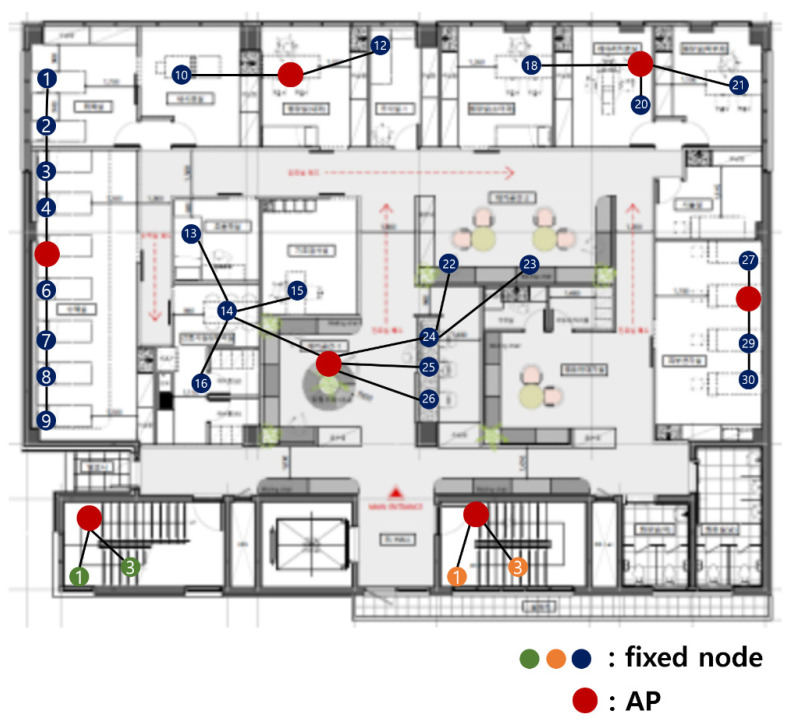
Result of applying 2-2 dividing step.

The final result of applying 2-2 dividing step to hospital network topology in Figure 4 is shown in Figure 11. A location of AP is set according to the cost reference in 2-1 dividing step.

## 4. Group Number-Based Network

In addition to fixed nodes, a hospital network has numerous sensor nodes with mobility. Doctors, nurses, patients, and caregivers continue to move their positions. They have their own communication equipment. Additionally, the medical staff carries tablets to handle electronic medical information. In this paper, group numbers are assigned to sensor nodes so that sensor nodes with mobility can connect to APs anywhere.

A group number enables data to be relayed to the AP through the other sensor nodes, even if direct connection to an AP is impossible. In a group number-based network, sensor devices with mobility are grouped based on the hop from AP. Then, it transmits data (only to the AP’s direction) to its next higher group. When the sensor node moves, and cannot transmit data to the origin sensor node of the next higher group, a new group number is assigned and data is transmitted using a new path.

### 4.1. Initial Group Number Setting

An initial group number setting is conducted when the network is first started. Algorithm 1 describes the process of the initial group number setting. The group number is assigned preferentially to fixed nodes and then assigned to nodes with mobility. An initial group number setting is performed in the following order:

1. An AP sets its group number to 0 and then transmits the group number information to fixed nodes within a distance that can be transmitted.
2. When a fixed node that has not set a group number receives the group number information, it adds 1 to the received group number and sets it as its own group number. When a fixed node that already has a group number receives the group number information, it compares its origin group number with the received group number. If the received group number is smaller than its origin group number, add 1 to the received group number and set it as its own group number. Thereafter, this new group number information is transmitted to other fixed nodes within a communication range.
3. Repeat step number two until the group numbers of all fixed nodes are set.
4. After group numbers are assigned to all fixed nodes, the group number assignment for the mobile sensors starts. Sensors with mobility receive a group number from the nearest AP or fixed node.
5. An AP transmits the group number information to the sensor node with mobility. When a moving node that has not set a group number receives the group number information, it adds 1 to the received group number and sets it as its own group number. Thereafter, this new group number information is transmitted to other moving nodes within a communication range.
6. Fixed nodes transmit group number information to the sensor node with mobility. When a moving node that has not set a group number receives the group number information, it adds 1 to the received group number and sets it as its own group number. When a moving node that already has a group number receives the group number information, it compares its origin group number with the received group number. If the received group number is smaller than its origin group number, add 1 to the received group number and set it as its own group number. Thereafter, this new group number information is transmitted to other moving nodes within a communication range.
**Algorithm 1** Initial group number setting 1: AP sets its group number to 0  2: AP transmits the group number information to nodes that in the communication range among fixed nodes ‘F = {$F_{1}$,$\;F_{2}$, …, $F_{n}$}’  3: while(! (all sensor node of F has group number)){ 4:  for *i* = 1, 2, …, *n*{ 5:    if($F_{i}$ has not set a group number){ 6:      $F_{i}$ set its group number as 1 7:      $F_{i}$ transmits the group number information to nodes that in the communication range among fixed nodes ‘F = {$F_{1}$,$\;F_{2}$, …, $F_{n}$}’ 8:     } 9:     else{ 10:       if(group number information < group number of $F_{i}\;$){ 11:         $F_{i}$ set its group number as (group number information + 1) 12:       } 13:     } 14:   } 15:  } 16: while(! (all mobile sensor nodes ‘M = {$M_{1}$,$\;M_{2}$ , …, $M_{m}$}’ has group number)){ 17:   for *i* = 1, 2, …, *m*{ 18:     if($\;M_{i}$ has not set a group number){ 19:       $\;M_{i}$ set its group number as 1 20:      $\;M_{i}$ transmits the group number information to nodes that in the communication range among mobile nodes ‘M = {$M_{1}$,$\;M_{2}$ , …, $M_{m}$}’ 21:     } 22:     else{ 23:       if(group number information < group number of $M_{i}$){ 24:        $M_{i}$ set its group number as (group number information + 1) 25:       } 26:    } 27:   } 28:  }*n*: the number of fixed sensor node *m*: the number of mobile sensor node

**Figure 12:**
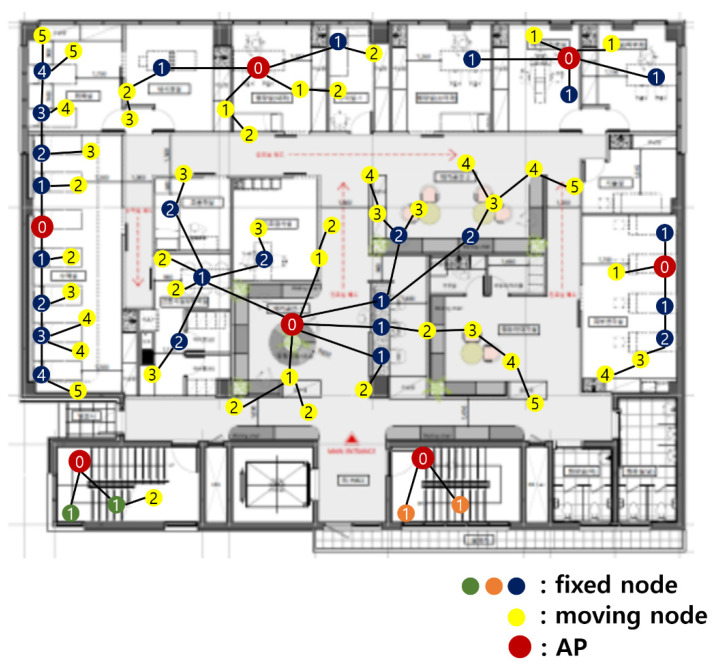
Result of the initial group number setting.

Figure 12 shows the result of the initial group number setting. The yellow circle represents the sensor nodes with mobility. The group number is written on each sensor node is a group number. The AP has the group number 0. A sensor node directly connected to the AP has the group number 1. The sensor node that relays data to the sensor node with group number 1 has group number 2.

### 4.2. Data Transmission Path

**Figure 13:**
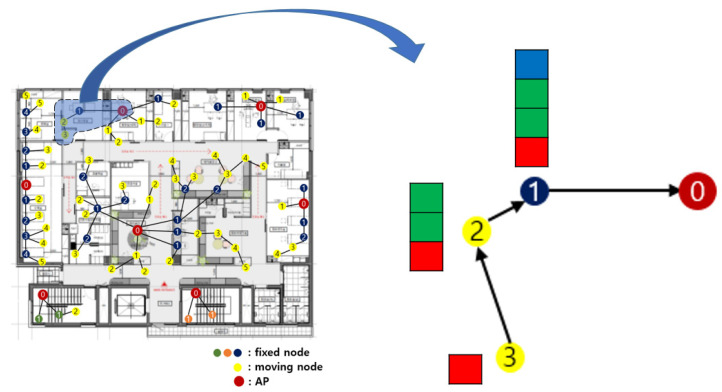
Data transmission path.

All sensor nodes relay their collected data to the sensor node with the next higher group number. Figure 13 is the enlarged part of Figure 12. The data that were collected by a sensor node with group number 3 are shown within the red boxes in Figure 13. It relays the data to a sensor node with group number 2, its feasible highest sensor node. The data that were collected by a sensor node with the group number 2 is shown in green boxes. It combines the data collected by itself and the data relayed from the sensor node having group number 3. Next, it forwards data to the sensor node having group number 1. The data that was collected by a sensor node with group number 1 is shown in the blue boxes. Finally, it combines the data collected by itself and the data relayed from the sensor node with group number 2 and delivers the data to an AP.

In the origin hospital network, in which medical sensors must be directly connected to an AP by one hop, data cannot be transferred to an AP if the mobile medical sensor is farther than the communication range of the AP. In the proposed network in this paper, even if a sensor node with mobility moves to another place, data can be continuously transmitted to the AP by changing group numbers. In a group number-based medical sensor network, not all sensor nodes directly deliver data to the AP. The network connectivity of sensor nodes is high because it relays data to other sensor nodes and to the AP. Rapid data transmission is possible even when direct connection to an AP is not possible.

### 4.3. Group Number Resetting

In a hospital sensor network, connectivity should be guaranteed wherever sensor nodes are located. If a sensor node moves over a certain distance or recognizes that data transmission to the upper group sensor is impossible, the group number should be reset. 

**Figure 14:**
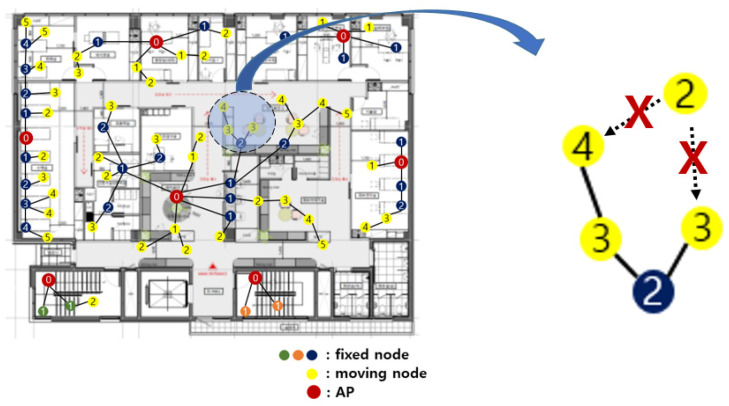
Group number resetting.

Figure 14 is an enlarged view of a part of Figure 12. A sensor node with group number 2 comes to a new location. It must relay data to another sensor node with group number 1. However, since there is no sensor node with group number 1 nearby, the data cannot be relayed. In this case, it reset the group number.

A sensor node that wants to reset group number requests group number information from another sensor node within its communication range. Upon receiving this request, the sensor node responds with its own group number information. The sensor node that wants to reset the group number collects the responses for a certain period of time. After a certain period of time, the tiniest group number information among the collected responses is selected. Then, the sensor node that wants to reset the group number adds 1 to the group number of the selected information and sets it as its own group number.

**Figure 15:**
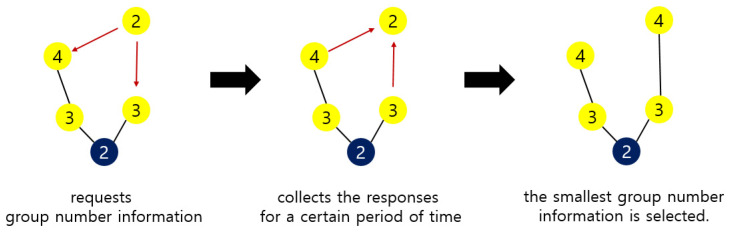
A process of group number resetting.

Figure 15 shows the group number resetting process of Figure 14. A sensor node requests the group number information of another sensor node within its communication range to reset the group number. The sensor nodes with the group numbers 3 and 4 respond to this. After collecting responses for a certain period of time, the smallest group number is selected. In Figure 15, group number 3 is selected. The sensor node adds 1 to this and resets 4 to its own group number.

## 5. Simulation

### 5.1. Comparison with the Method to Check All Cases 

To achieve an optimal AP deployment way it is required to compare the number of all cases. Therefore, in order to verify the validity of the method proposed in this paper, we compared it with a method that compares the number of all cases by simulation.

For simulation, we used Matlab and the parameters for Equation (1) are set as (2). Five simulations were performed for 15, 16, 17, and 18 sensor nodes. Each time it was run, the shape of the network topology was different. For each sensor node topology, 5 results were averaged.

Table 4 shows the time spent to obtain the AP layout. It is compared by considering all cases and the proposed method in this paper. Considering all cases consumes much more time than the method proposed in this paper. Considering all cases consume about 4.42 h, on average, to obtain the AP layout for 18 sensor nodes, while the method proposed in this paper consumes about 15 s. In the case of Figure 9, with 30 sensor nodes, considering all cases would take about 10 years. It is impossible to use this in the real world. The method proposed in this paper consumes about 24 s to obtain the AP layout for 30 sensor nodes.

**Figure 16:**
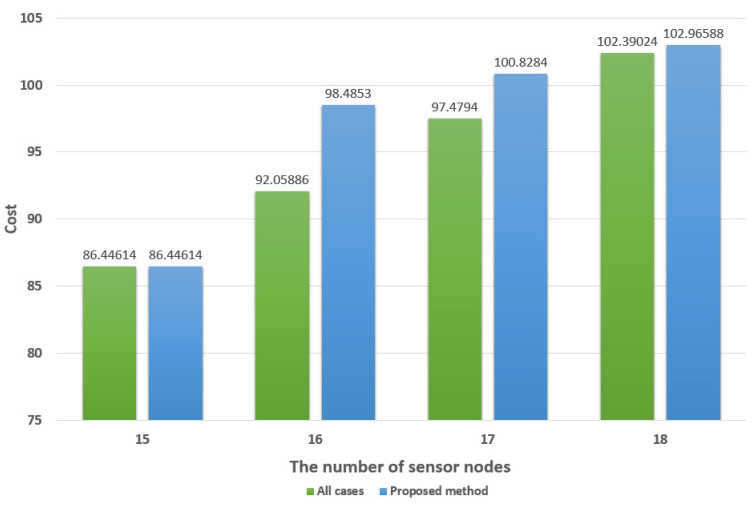
The cost comparison between all cases and the proposed method.

Figure 16 shows the average installation cost for Table 4. This is the result obtained by applying *a* = 10, *b* = 3 to Equation (1). There is no significant difference in the installation costs between the consideration of all cases and the method proposed in this paper.

As a result, the method proposed in this paper can obtain the AP layout by consuming a short amount of time, rather than considering all cases; the AP layout does not differ significantly, in terms of the installation cost. Hospitals often have to move medical equipment or computers with fixed properties. In order to obtain an optimal AP layout for a constantly changing network, it is necessary to obtain it quickly.

### 5.2. Comparison with the Genetic Algorithm

The Genetic Algorithm is utilized to obtain an optimized result, which has been steadily studied before. For example, the Genetic Algorithm-Based Self Organizing Network Clustering (GASONeC) uses the Genetic Algorithm to find the most optimal cluster header among sensor nodes. The GASONeC uses the distance between the two sensor nodes and the number of sensor nodes to calculate fitness fuction [24]. The GASONeC can be applied to find the optimal AP deployment. The GASONeC is simple to apply and to be used to solve optimal problems. However, the results obtained using this method are not reliable. Because this algorithm is based on probability, the result is different each time it is applied. It cannot be assured that there will not be a better case than the result obtained. 

**Figure 17:**
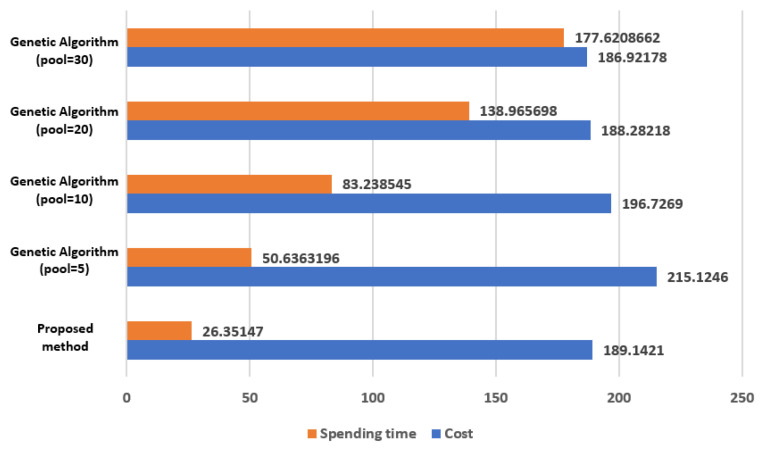
The comparison results in computation time and cost between the Genetic algorithm and the proposed method.

We compare the method proposed in this paper with the Genetic Algorithm. It targets 30 sensor nodes. Figure 17 shows the comparison results. The method proposed in this paper is the result obtained by applying *a* = 10, *b* = 3 to Equation (1). The Genetic Algorithm is the result obtained by setting the crossover probability = 0.8, mutation probability = 0.006, and 100 iterations. The Genetic Algorithm results vary, according to the setting values set for the initial pool and, in this simulation, the number of pools was set to 5, 10, 20, and 30. Since the Genetic Algorithm shows different results each time it is executed, Figure 17 is shown as the average value, by experimenting 5 times per pool.

In Figure 17, the cost of the method proposed in this paper is less than that of the Genetic Algorithm or there is no significant difference. On the other hand, the computation time is remarkably short.

**Figure 18:**
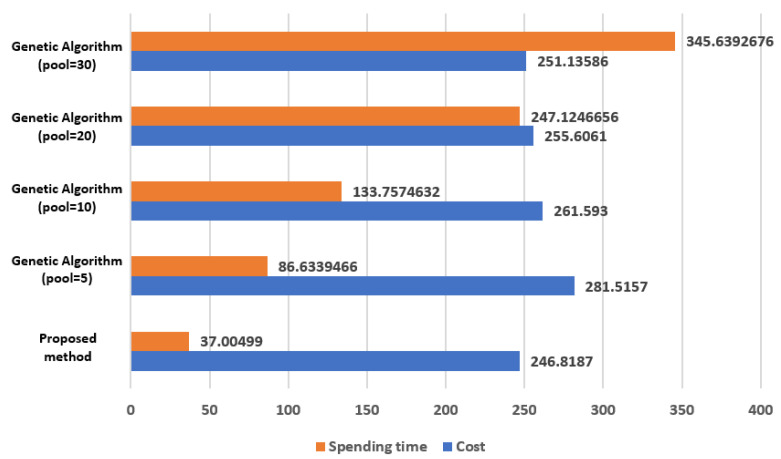
The resulting comparisons between the Genetic Algorithm and proposed method in terms of the computation time and cost of 40 sensor nodes.

Figure 18 is the result of comparing 40 sensor nodes. The method proposed in this paper is the result obtained by applying *a* = 10, *b* = 3 to Equation (1). The Genetic Algorithm is the result obtained by setting the crossover probability = 0.8, mutation probability = 0.006, and 100 iterations. The Genetic Algorithm results vary, according to the setting values set for the initial pool, and in this simulation, the number of pools is set to 5, 10, 20, and 30. Since the Genetic Algorithm shows different results each time it is executed, Figure 17 is shown as the average value, by experimenting 5 times for each pool.

When the method proposed in this paper is applied, the cost is less than that of the Genetic Algorithm. Additionally, the computation time is remarkably short.

When the Genetic Algorithm is applied, a different result is displayed each time it is executed. Therefore, in order to apply the Genetic Algorithm, it is necessary to run it several times by adjusting the crossover probability, mutation probability, iteration, and the number of initial pools. The process of adjusting these values is time consuming, but it cannot be guaranteed that the execution result is optimal. Therefore, the simulation comparison results of this paper do not include the time to adjust the crossover probability, mutation probability, iteration, and the number of initial pools. On the other hand, since the method proposed in this paper shows the same result every time it is applied, it is possible to guarantee the optimal result.

## 6. Conclusions

In this paper, we propose a method for making stable hospital network operations. A hospital is a network that has sensor nodes both with and without mobility. Since sensor nodes in hospitals are always in an environment where power can be supplied, it is more important to have stable network connectivity rather than to use energy efficiently. In this paper, we propose a method to obtain an AP layout that minimizes the overall network operation cost by spending a small amount of time. Additionally, a group number is assigned to each sensor node so that network connectivity can be guaranteed at any time, no matter where the mobile sensor nodes move.

## Figures and Tables

**Table 1:** Packet loss rate.

Distance-Obstructions	Packet Lost Rate
3 m	0.13%
5 m	0.19%
5 m-1door	0.32%
10 m	0.85%
10 m-1door	1.08%
10 m-2doors	1.23%
moving patient	20.15%

**Table 2:** Combinations of the blue part.

Before Applied			After Applied	
	Combinations	Cost	Applied Combinations	Applied Cost
**1**	(8, 5, 4, 4, 4, 3, 1, 1)	184	(9, 5, 5, 4, 4, 3)	187
**2**	(8, 8, 4, 4, 3, 3)	186	(10, 9, 4, 4, 3)	188
**3**	(8, 8, 7, 3, 2, 1, 1)	189	(9, 8, 7, 3, 2, 1, 1)	201

**Table 3:** Combinations of the green part.

Before Applied			After Applied	
	Combinations	Cost	Applied Combinations	Applied Cost
**1**	(3)	20	(3)	20
**2**	(2, 1)	25	(2, 1)	25

**Table 4:** Time spent to obtain AP layout.

		The Number of Sensor Nodes							
		15		16		17		18	
		All Cases	Proposed Method	All Cases	Proposed Method	All Cases	Proposed Method	All Cases	Proposed Method
**Number of simulation runs**	**1**	1395.777	5.567255	3346.086	7.838274	7030.184	11.45018	16,088.71	14.1234
	**2**	1356.374	6.392298	3065.544	8.11234	6912.407	12.4214	15,885.37	14.1256
	**3**	1403.543	6.109328	3412.523	7.9934	7123.94	12.15124	15,923.11	15.92189
	**4**	1389.235	7.1143	3387.412	8.0124	7111.85	11.94673	15,488.22	15.1235
	**5**	1394.432	6.9932	3314.324	8.412354	6899.523	10.93876	16,183.2	15.7315
**Average (s)**		1387.872	6.435276	3305.178	8.073754	7015.581	11.78166	15,913.72	15.00518
